# The More You Know, the Less You Stress: Menstrual Health Literacy in Schools Reduces Menstruation-Related Stress and Increases Self-Efficacy for Very Young Adolescent Girls in Mexico

**DOI:** 10.3389/fgwh.2022.859797

**Published:** 2022-04-14

**Authors:** Jeanne L. Long, Jacquelyn Haver, Pamela Mendoza, Selvia M. Vargas Kotasek

**Affiliations:** ^1^Department of Education and Child Protection, Save the Children, Washington, DC, United States; ^2^Programs, Save the Children, Mexico City, Mexico

**Keywords:** menstrual health literacy, very young adolescents, stress, self-efficacy, Mexico, puberty, school health

## Abstract

Improving the menstrual health literacy of girls and boys is a key strategy within a holistic framework of Save the Children's school health and comprehensive sexuality education programming. As menstrual health is an emerging area of study and programming, Save the Children continues to learn and adjust its interventions using program evaluations and rigorous monitoring. This paper will examine program-monitoring data from three cohorts, representing 47 public schools in Mexico City, Puebla, and Mérida, Mexico. The study focuses on female students in 5th and 6th grade who participated in We See Equal, a school-based program centered on gender equality and puberty education, between September 2018 and December 2019. This study used a cross-sectional quantitative cohort approach to document changes in girls' experiences and perceptions around managing menstruation in school. The analysis compares girls' knowledge and experiences before and after participation in We See Equal to understand how knowledge changes over the program and how those changes may contribute to menstruation-related school engagement, stress, and self-efficacy (MENSES) outcomes. Multivariate regression models explored relationships between MENSES outcomes, knowledge and socioeconomic status (SES). Overall, results show that the more knowledge girls acquired, the higher their self-efficacy score and the lower their stress score, however, certain MHH knowledge was more predictive of MENSES outcomes and varied by SES. Among girls from lower SES, we observed significant relationships between knowing what their period was prior to menarche and the three MENSES outcomes. Decreases in menstruation-related stress were driven by items related to the practical knowledge of how to dispose of sanitary pads and reduced feelings of nervousness on days they had their period at school. Increases in self-efficacy were primarily driven by girls' confidence in their ability to track their period from month to month, feelings that they could still do well on an exam if they had their period at school, and security that they could ask a friend to lend them a pad if they needed one. Implications for future menstrual health literacy programming and targeting populations for menstrual health education, as well as priorities for future research will be discussed.

## Introduction

Menstrual health and hygiene (MHH) is gaining attention as a research topic and program intervention for adolescent girls (1). Marking the beginning a woman's reproductive life, menstruation represents a critical point to influence girls' education, health and reproductive outcomes (2). Early menarche has been associated with increased risk of adolescent pregnancy and increased reports of being unhappy at home (3). Menarche is a particularly salient experience for girls, as the onset of menses is often unexpected, uncomfortable and visible. Over a decade of formative research in low-middle income countries (LMIC) across the world has documented girls' hardships managing their periods in school and the negative emotions that accompany their menstrual period: feelings of anxiety, stress, confusion and stigma regarding their period (4, 5). A lack of information and practical guidance about menstruation, poor water, sanitation and hygiene infrastructure in schools, limited access to menstrual materials, and unsupportive social environments that force girls to contend with teasing, secrecy, and social norms that dictate a range of behavioral restrictions contribute to the negative emotions experienced by girls during menses (6–18). Socioeconomic status contributes to worse experiences managing menstruation, as girls and women from poorer households may lack space, soap and water to manage their menses (19), and may be at increased risk of sexual violence as girls attempt to meet their menstrual needs (20). In 2020, it is estimated that 44.4% of the total female Mexican population lives in poverty and 19.5% lack basic household services like drinking water and access to showers (21), adding up to 27 million women who potentially face an obstacle to menstrual hygiene. On average 611 incidents of family violence are reported daily in Mexico, 160 alleged victims of malicious injuries, 46 alleged victims of rape, and 10 murders (femicides and homicides), with evidence of gender-based violence increasing during the pandemic (22).

Evidence suggests the need to focus efforts on very young adolescents (ages 10-14), as earlier interventions may improve gender-based violence, adolescent pregnancy, early marriage and school dropout outcomes (23, 24). Instilling healthy behaviors and habits earlier in life could result in greater impacts for years to come (25–28). UNESCO suggests initiating comprehensive sexuality education before puberty, within primary education (29). Unfortunately, caretakers and teachers often feel ill equipped to deliver this information to children (30). MHH literacy fits squarely within a comprehensive sexuality education (CSE) and puberty education, but is often poorly taught, not included in teacher training, and although these elements are typically part of the basic education curricula, they are not assessed (1, 31). In Mexico, the SEP offers a plethora of teacher continuing education courses focused on topics such as incorporating gender into the classroom, educating children on healthy lifestyles, and healthy development of students (32). However, these potentially valuable offerings are not required, and depend on each teacher's ability and interest to partake in them.

Studies evaluating the impacts of MHH education on school attendance have yielded mixed results (33–38), with different countries and programs demonstrating varying levels of absenteeism (39–41) and coping strategies among menstruating girls (42). Alternatively, in qualitative studies, girls consistently elaborate on the struggles of managing their periods at school. Girls' self-reports of reduced academic and school participation, fear and distraction (11, 13), self-isolation or social exclusion, missing class, and leaving school during the day (9, 10, 18, 40, 41) demonstrate the need to evaluate the impact of girls' menstruation experiences in school beyond absenteeism (43, 44). Consequently, studies are examining the linkages between menstrual health, menstrual health knowledge, sanitation, education, and psychosocial outcomes (30, 42, 45–48). Psychosocial elements of menstrual health may be a more relevant indicator than attendance for the Mexican context. Attendance rates are high through 14 years of age (93%), with noticeable declines starting at age 12 and dropping to 73% by age 15 (49). Though enrollment rates remain high in early schooling, 5th grade is when the greatest dropouts occur in primary grades (50). Education as a protective factor for other health outcomes is well known and relevant in Mexico. With the highest pregnancy rate among OECD countries, the rate more than doubles (16-39%) when disaggregated by school enrollment status among out of school adolescent girls (51). Menstrual health is not well studied in Mexico, but what is taught through the education system may have important ramifications, as Mexico grapples with high rates of adolescent pregnancy, high prevalence of early sexual debut (52) and gender-based violence (22, 53–55), all of which are higher among girls who are out of school. Intervening early to improve skills-based MHH literacy within a CSE framework, could be an important catalyst to increase girls' confidence in early adolescence and improve future health and education outcomes.

## Program Implementation

We See Equal (WSE) is a school-based intervention for very young adolescents (VYA) with the objective of increasing knowledge of physical, emotional and social changes associated with puberty, as well as increasing positive gender attitudes centered on themes of respect, equality and empathy (56). WSE was designed in accordance with the Focusing Resources on Effective School Health (FRESH) Framework (56). The Choices curriculum was included as the evidence-based education component (57). Choices in Mexico was enhanced both in the structure and content, serving as a resource for teachers to use in the classroom and adapted to address the nuances of gender inequality for VYA in project locations. Additionally, Choices in Mexico integrated three puberty and menstrual health lessons aligned with curricular requirements of the Secretariat of Public Education (SEP) in Mexico (58). The final Choices curricula in Mexico consisted of eleven total lessons focusing on the following themes: Puberty Knowledge and Menstruation (see Table 1), Gender Equality, Respect, Empathy, and Empowerment (59). By December 2019, WSE had trained 413 teachers (343 women and 70 men) and reached 10,132 children (5,026 girls and 5,106 boys) in 47 public schools in Mérida, Mexico City, and Puebla, Mexico.

**Table 1:** Session objectives of puberty and menstruation lesson plans.

**Lesson name**	**Objective**
Getting to know puberty	Identify the physical, emotional, and social changes experienced during puberty
Getting to know my body	Explain the biological processes and characteristics of menstruation and ejaculation.
Taking care of my body	Identify important puberty-related hygiene habits
Who is who?	Understand the differences between biological characteristics that refer to sex and those that are socially constructed and link to gender.

A team of eight school health promoters, one project lead and one part-time monitoring and evaluation staff implemented Choices in Mexico City, Puebla City and Mérida. Program inputs included an adapted Choices teacher manual (59), staff training, teacher training, 11 teacher-led Choices lesson in 5th and 6th grade classrooms, extra-curricular student equality clubs and water, sanitation and hygiene (WASH) school improvement plans. Choices teacher training and classroom implementation were implemented over one school semester. Each Choices lesson lasted approximately 1 hour and was structured to include a review of key messages from the previous lesson, a central lesson, and space for reflection. Energizers and relaxation exercises were incorporated throughout lessons. Teachers were encouraged to create ground rules during the first session that would foster trust and respect among students, and to display their ground rules before every Choices session. A question box was set up in each classroom so that students could ask questions anonymously after each session.

Teacher training and Choices session were organized in three blocks so that teachers were trained on 3-4 sessions at a time and then implemented those sessions in the classroom prior to the next training. This allowed program staff to collect feedback from teachers on the success of implementation. Promoters delivered three 4-h teacher trainings with 15-25 teachers per session. Trainings included practical application of Choices, as well as the theoretical underpinnings of the curricula related to child rights, adolescent sexual reproductive health and gender. Promoters observed at least one in-class lesson per trained teacher to monitor fidelity of instruction. The WSE program was funded by Procter & Gamble.

This paper will explore program monitoring data collected at program baseline and endline across three cohorts to identify trends that suggest providing menstrual health and puberty education in schools can improve girls' experiences managing their periods at school, reducing stress and increasing feelings of self-efficacy. This analysis aimed to answer the following research questions: (1) To what extent does menstrual health and hygiene (MHH) interventions contribute to increased menstruation-related engagement and self-efficacy and reduced menstruation-related stress (MENSES)? (2) How do MENSES outcomes and MHH knowledge differ by socioeconomic status? Does WSE narrow those gaps? (3) What is the relationship between MHH knowledge levels and MENSES outcomes?

## Methods

This study used a cross-sectional quantitative cohort approach to document changes in girls' experiences and perceptions around managing menstruation in schools. A random sample of girls and boys was derived from all 5th and 6th grade rosters of participating schools in Mexico City, Puebla and Mérida. The baseline and endline assessments included a paper-based true/false knowledge assessment that was linked to specific students, as well as a digital knowledge, attitudes and practices (KAP) survey performed by enumerators that assessed boys and girls KAP for key themes of We See Equal. Digital surveys were read aloud by trained enumerators and collected *via* tablets using KoBo Toolbox. Each child assessed was assigned a numeric code to de-identify participants from personal data and to link baseline and endline results for analysis.

This study focuses on 140 female students at baseline and 193 at endline from all 47 schools across three semester cohorts between September 2018 and December 2019. The analysis examines a subset of menstruation and puberty-specific questions among girls who self-reported to have reached menarche and had managed their periods while in school. A pre-and post-test approach with no comparison group tracked trends in girls' menstruation-related school engagement, stress, and self-efficacy (MENSES). The analysis combined three cohorts to increase statistical power and to disaggregate data by socioeconomic status (SES) and knowledge levels.

MENSES item responses measured frequency (almost always, sometimes, and never), strength (a lot, a little, and never), and agreement (strongly agree, agree, disagree, and strongly disagree) with girls' school engagement, feelings and beliefs, respectively. Scales were converted from ordinal scales to percentage scales to simplify interpretation over time and across subgroups.

For this analysis, responses were coded on a scale of 0-2 for the participation and stress domain, and the self-efficacy was coded on a scale of 0-3. For the participation and self-efficacy domain, a number closer to 100% implies more participation or confidence. For the stress domain, a number closer to 0% means that girls were feeling less worried while doing certain activities at school.

For the knowledge questions, girls responded whether they have learned about puberty and menstruation at school (yes, a little, and no). For this analysis, “yes” and “a little” responses were combined as we are interested in whether they have acquired this knowledge at school and how knowledge has changed over time; converting this ordinal scale simplifies the analysis and interpretation. Finally, the socioeconomic status was calculated using the question “having a computer at your house” as a proxy. This was the only SES-related question included in the WSE KAP survey.

Data analysis was conducted using statistical software Stata 17. Summary statistics from the analysis are presented to display girls' knowledge and MENSES outcomes. Multivariate regression models were used to explore relationships between MENSES outcomes and girl's knowledge and other sociodemographic characteristics. We fit three Ordinary Least Squares regression models, adjusting the standard errors for clustering girls at the school level.


$$\begin{array}{l} \text{Model }1:\;Y_{ij}=\;\alpha +\;\beta_{1}K_{ij}+{\beta_{2}SES}_{ij}+\;\beta_{3}Coh_{ij}+Z_{j}^{\prime }+\;\epsilon_{ij} \end{array}$$

*Y*_*ij*_ represents each MENSES outcome (engagement, stress, and self-efficacy) for girl *i* in school *j*, *K*_*ij*_ represents the aggregated knowledge questions (including all five questions) for girl *i* in school *j*, *SES* represents the SES index measured as owning a computer, *Coh*_*ij*_ represents the implementing cohort for girl *i* in school *j*, and $Z_{j}^{\prime }$ is a vector of school-geographic variables. The error term ϵ_*ij*_ consists of unobserved girl characteristics.


$$\begin{array}{l} \text{Model }2:\;Y_{ij}=\;\alpha +\;\beta_{1}X_{ij}+{\beta_{2}SES}_{ij}+\;\beta_{3}Coh_{ij}+Z_{j}^{\prime }+\;\epsilon_{ij} \end{array}$$

*Y*_*ij*_ represents each MENSES outcome (engagement, stress, and self-efficacy) for girl *i* in school *j*, *X*_*ij*_ represents knowledge of what the period was when had it for the first time for girl *i* in school *j*, *SES* represents the SES index measured as owning a computer, *Coh*_*ij*_ represents the implementing cohort for girl *i* in school *j*, and $Z_{j}^{\prime }$ is a vector of school-geographic variables. The error term ϵ_*ij*_ consists of unobserved girl characteristics.


$$\begin{array}{l} \text{Model }3:\;Y_{ij}=\;\alpha +\;\beta_{1}A_{ij}+{\beta_{2}SES}_{ij}+\;\beta_{3}Coh_{ij}+Z_{j}^{\prime }+\;\epsilon_{ij} \end{array}$$

*Y*_*ij*_ represents each MENSES outcome (engagement, stress, and self-efficacy) for girl *i* in school *j*, *A*_*ij*_ represents having a trusted adult to talk about puberty for girl *i* in school *j*, *SES* represents the SES index measured as owning a computer, *Coh*_*ij*_ represents the implementing cohort for girl *i* in school *j*, and $Z_{j}^{\prime }$ is a vector of school-geographic variables. The error term ϵ_*ij*_ consists of unobserved girl characteristics.

Statistical significance is defined in line with social science research standards at the probability of rejecting the null hypothesis due to random sampling error <5%. Note that, except where explicitly noted, statistical significance does not indicate a causal link between two variables; it means only that there is an observed relationship. Additionally, some of the results are disaggregated by socioeconomic status and knowledge levels.

Tool and sample are described in detail in the following sections.

### Tool

The Menstruation-related Engagement, Self-Efficacy and Stress (MENSES) assessment aims to measure girls' experiences managing their menses at school (60, 61) using 45 questions across three domains, or outcomes: school engagement, stress, and self-efficacy. The tool was adapted to the Mexico context and translated sensitively to ensure that the meaning of the questions remained the same as in previous pilots in differing country contexts.

#### Engagement

The engagement domain covers school participation experiences during menstruation. Girls are asked to think about behaviors they may or may not have done the last time they had their period at school, such as raising a hand or volunteering to answer a question if the answer is known, engaging in school-based social activities, classroom concentration, and missing class time for menstrual management.

#### Stress

The stress domain probes on menstrual related fears or worries a girl may have experienced the last time she managed her menstruation while at school. The questions ask about nervousness and worries related to experiencing a stain, teasing from classmates, menstrual pain, using the bathroom, having access to water, and ability to dispose of used menstrual materials. The section also inquires about feelings of loneliness during menstruation.

#### Self-Efficacy

The self-efficacy domain measures girls' beliefs in their own ability to do a menstrual-related task or school activity when menstruating at school. The questions include ability to request support from teachers or peers, gaining access to the school bathrooms, disposing of a sanitary material, accessing menstrual materials if needed, or standing up for herself or a friend if being teased about menstruation. The questions also cover confidence to perform well academically during menses.

#### Puberty and Menstruation

Puberty and menstruation questions in the digital KAP survey were primarily used for monitoring (i.e., have you learned about menstruation in school?) and as filters for MENSES questions. Boys and girls who had not begun their menses were asked other menstruation knowledge questions not captured in this analysis.

### Sample

Schools were purposely selected in Puebla, Mérida, and Mexico City, in coordination with local education officials. A total of 900 girls were surveyed at baseline from 47 schools across three semester cohorts between September 2018 and December 2019. At endline, data was collected from as many original participants as could be found in the selected schools. This sample is a random representative sample of girls from these 47 schools. Out of 900 total girls, 140 girls at baseline and 193 girls at endline reported having their period at school. In Table 2, we present the study sample characteristics for the girls who had their period at school at the interview. At baseline, 39% of the girls were from Merida, 26% were from Mexico City, and the rest were from Puebla. At baseline, half of the sample (50%) reported having a computer at home and most of the sample (77%) were in Grade 6. At endline, more girls were surveyed, as more girls reached menarche over the course of the school semester.

**Table 2:** Study sample by location, grade and SES at baseline and endline.

**Characteristics**	**Baseline *N* = 140 Number *n*(%)**	**Endline *N* = 193 Number *n*(%)**
**Location**		
Merida	55 (39)	75 (39)
Mexico City	37 (26)	56 (29)
Puebla	48 (34)	62 (32)
**Grade**		
5th	32 (23)	27 (14)
6th	108 (77)	166 (86)
**SES**		
Own a computer (yes)	69 (50)	101 (52)

### Ethics Considerations

The study was submitted to the Save the Children US Ethics Review Committee, the assigned reviewer granted final approval in June 21, 2018, #FWA00022738. Caregiver signed consent and verbal assent was obtained by children prior to starting the survey questionnaire.

## Results

Menstruation-related stress and self-efficacy outcomes improved after completion of the WSE program at the p < .001 level. Girls experienced a 6% reduction in stress the last time they had their period in school and a 6% increase in their confidence of their own ability to manage their periods in school (see Table 3). Table 4 shows that decreases in stress were primarily driven by reductions in worries about how to dispose of their sanitary pads (−9pp), feelings of nervousness while menstruating at school (−8 pp), and concerns that their classmates would talk about them having their period (−9 pp). Increases in self-efficacy were most influenced by girls' confidence to track their own period to know when it's coming next (+16pp), feelings that they could perform equally as well on an exam on days when they had their period (+12 pp), and the confidence that they could ask a friend to lend them a sanitary pad (+10 pp).

**Table 3:** Comparison of baseline and endline changes in MENSES outcomes.

	**Baseline *N* = 140 %**	**Endline *N* = 193 %**
**MENSES** **Sample: Girls that have had their period at school**	79	81
Engagement		
Stress^***^	68	62
Self-efficacy^***^	75	81

* *p < 0.05*,

** *p < 0.01*,

*** *p < 0.001*.

**Table 4:** MENSES scores and most improved items over time^a^.

**MENSES items**	**Baseline N = 140 %**	**Endline N = 193 %**	**Change pp**
**Engagement**	79	81	2
Participated in a physical education class	71	75	4
Felt like leaving school during the school day to change your sanitary pad	33	29	−4
Had a difficult time paying attention to the teacher because you were thinking about your period^*^	33	24	−9
Stress^***^	68	62	−7
Worried that their girlfriends would gossip about them^**^	65	56	−9
Felt nervous^**^	74	65	−8
Worried about how to dispose sanitary pad at school^**^	65	55	−9
Self-efficacy^***^	75	81	6
Ask a friend to borrow a sanitary pad^***^	73	83	10
Do well on an exam^***^	78	90	12
Track your period to know what day your next period is coming^***^	53	69	16

* *p < 0.05*,

** *p < 0.01*,

*** *p < 0.001*.

a *MENSES items are provided in Supplementary Materials*.

General knowledge regarding puberty and menstruation in this population was high at baseline. Table 5 shows the change in MHH and puberty knowledge among menstruating girls and the entire female WSE sample. The majority of menstruating girls knew what puberty was (80%) and had learned about both puberty and menstruation at school before the start of WSE, 94 and 88%, respectively. At endline, almost all girls obtained this basic knowledge, with statistically significant increases in the percentage of girls who knew of puberty (15%), learned about it in school (5%) and learned about menstruation in school (10%). At endline, 65% girls responded to knowing more (a lot/a little) in the five knowledge questions considered for this analysis compared to 47% girls at baseline (Table 6). Among all girls surveyed (*n* = 900), puberty and MHH knowledge increased significantly across all five items (Table 5). The largest jumps were knowing what puberty was (24%), having a trusted adult they could ask about puberty (24%), and learning about puberty at school (28%).

**Table 5:** Comparison of baseline and endline changes in MHH literacy.

	**Baseline *N* = 900 *n* (%)**	**Endline *N* = 883 *n* (%)**
**Menstrual health and hygiene literacy**		
**Sample: All girls interviewed at baseline**		
Girls who know what puberty is^***^	619 (70)	819 (93)
Girls who trust and adult to ask about puberty^***^	573 (66)	773 (88)
Girls who learned about puberty at school^***^	572 (66)	811 (92)
Girls who know what menstruation is ^***^	802 (90)	849 (96)
Girls who learned about menstruation at school^**^	631 (71)	672 (77)
	**Baseline** ***N*** **=** **140** ***n*** **(%)**	**Endline** ***N*** **=** **193** ***n*** **(%)**
**Menstrual health and hygiene literacy**		
**Sample: Girls that have had their period at school**		
Girls who know what puberty is^***^	112 (81)	183 (95)
Girls who trust and adult to ask about puberty	105 (94)	175 (95)
Girls who learned about puberty at school^**^	105 (94)	184 (99)
Girls who know what menstruation is	140 (100)	193 (100)
Girls who learned about menstruation at school^**^	122 (88)	159 (98)
Girls who knew what menstruation was when they had their first menstruation	101 (74)	153 (80)

* *p < 0.05*,

** *p < 0.01*,

*** *p < 0.001*.

**Table 6:** Percentage of girls that responded a little/a lot to knowledge questions.

**No. of questions**	**Baseline**	**Endline**
0	11%	2%
1	7%	2%
2	15%	6%
3	11%	4%
4	10%	21%
5	47%	65%

Girls from high and low socioeconomic groups demonstrated statistically significant improvements in stress and self-efficacy (Table 7), and MHH knowledge (Table 8) after WSE. At baseline, low SES girls reported significantly higher levels of menstruation-related stress than high SES girls (5pp). After WSE, low SES girls still had higher stress levels, but the difference was no longer statistically significant and they experienced a 1pp higher improvement in stress levels compared to their high SES peers. Conversely, differences between self-efficacy among the two SES groups were not statistically significant and we observed statistically significant improvements over time among high and low SES girls, 6 and 5pp, respectively.

**Table 7:** Changes in MENSES Outcomes between baseline and endline by SES.

**Outcome**	**SES**	**Baseline *N* = 140 %**	**Endline *N* = 193 %**	**Change over time pp**
Participation	Low	77	79	2
	High	81	83	2
	Gap high-low (pp)	4	4	
Stress	Low	71	64	−7^***^
	High%	66	60	−6^***^
	Gap high-low (pp)	−5^*^	−4	
Self-efficacy	Low	74	79	5^***^
	High	77	83	6^***^
	Gap high-low (pp)	3	4	

* *p < 0.05*,

** *p < 0.01*,

*** *p < 0.001*.

**Table 8:** Change in MHH knowledge by socioeconomic status among all female WSE survey respondents.

	**Low SES**			**High SES**		
	**Baseline N = 452 %**	**Endline N = 418 %**	**Change over time pp**	**Baseline N = 440 %**	**Endline N = 464 %**	**Change over time pp**
Do you know what puberty is?	64	88	24^***^	76	94	18^***^
Do you trust an adult to ask about puberty?	92	92	0	94	96	2
Have you learned about puberty in school?	92	99	7^***^	94	99	5^**^
Do you know what menstruation is?	77	93	16^***^	83	95	12^***^
Have you learned about menstruation at school?	77	96	19^***^	84	98	14^***^

* *p < 0.05*,

** *p < 0.01*,

*** *p < 0.001*.

Trends in MHH knowledge differed across SES groups. Results for all girls surveyed highlight that while high SES girls had greater awareness of these topics at baseline and endline, the knowledge gaps for low SES girls reduced in all knowledge questions, except having a trusted adult they could talk to about puberty. In particular, Table 8 shows that compared to high SES girls, low SES girls demonstrated the largest increases in knowing about puberty (18 vs. 24% change) and having learned about menstruation at school (14 vs. 19% change).

MENSES outcomes were examined by level of MHH knowledge at baseline and endline (Table 9). Prior to the WSE program, girls with lower MHH knowledge demonstrated statistically significant lower MENSES outcomes compared to their peers with more MHH knowledge. At endline, the gaps narrowed for engagement and stress, though differences between girls with high and low knowledge were no longer statistically different. However, for menstruation-related self-efficacy the gap widened between low and high knowledge girls at endline, increasing from 3 to 7 pp, suggesting that girls that had already obtained puberty and MHH information boosted their confidence on managing menstruation at schoolm. These relationships were further examined among only low SES girls (Table 10) and similar trends emerged, except that at baseline the engagement and stress gaps were slightly wider between those with higher and lower knowledge (8 pp) and differences in self-efficacy were non-significant. Similar to the aggregate group, at endline, girls with higher knowledge had significantly higher self-efficacy and the differences in other MENSES outcomes were not significant.

**Table 9:** MENSES domains by level of knowledge at Baseline and Endline.

**MENSES domains**	**Baseline**				**Endline**			
	**Low knowledge N = 51 %**	**High knowledge N = 89 %**	**Gap (high-low knowledge) pp**	**Significant difference (low vs. high)**	**Low knowledge N = 53 %**	**High knowledge N = 140 %**	**Gap (high-low knowledge) Pp**	**Significant difference (low vs. high)**
Engagement	75	81	6	^*^	77	82	5	
Stress	74	67	−7	^*^	67	62	−5	
Self-efficacy	73	76	3	^*^	75	82	7	^**^

* *p < 0.05*,

** *p < 0.01*,

*** *p < 0.001*.

**Table 10:** MENSES domains for girls with low SES by level of knowledge over time.

	**Girls with low SES—Baseline**				**Girls with low SES—Endline**			
	**Low knowledge N = 33 %**	**High knowledge N = 36 %**	**Gap (high-low knowledge) pp**	**Statistical difference (low vs. high)**	**Low knowledge N = 31 %**	**High knowledge N = 61 %**	**Gap (high-low knowledge) pp**	**Statistical difference (low vs. high)**
Engagement	72	80	8	^*^	77	80	3	
Stress	76	68	−8	^*^	67	64	−3	
Self-efficacy	73	75	2		74	80	6	^*^

* *p < 0.05*,

** *p < 0.01*,

*** *p < 0.001*.

**Figure 1:**
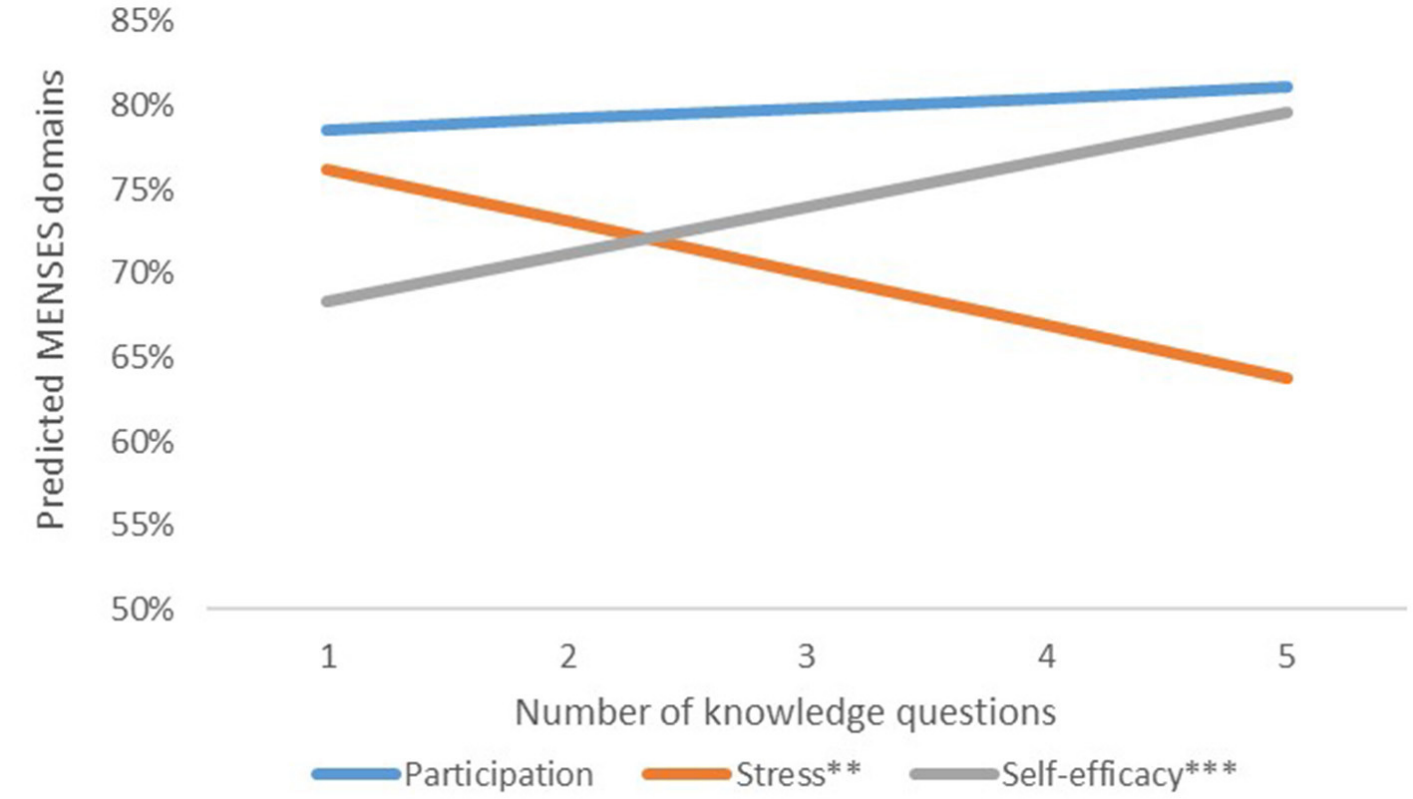
Association between menstrual health and puberty knowledge and MENSES outcomes. ^*^p < 0.05, ^**^p < 0.01, ^***^p < 0.001.

Multivariate regression models controlling for region and cohort explored relationships between MENSES outcomes, MHH knowledge and SES. Regression analyses shows that aggregated knowledge (including all five questions) is significantly negatively associated with stress and positively associated with self-efficacy at baseline and endline. SES was significantly positively associated with self-efficacy. In sum, the more knowledge girls acquired, the higher their self-efficacy score and lower their stress score (Figure 1).

**Figure 2:**
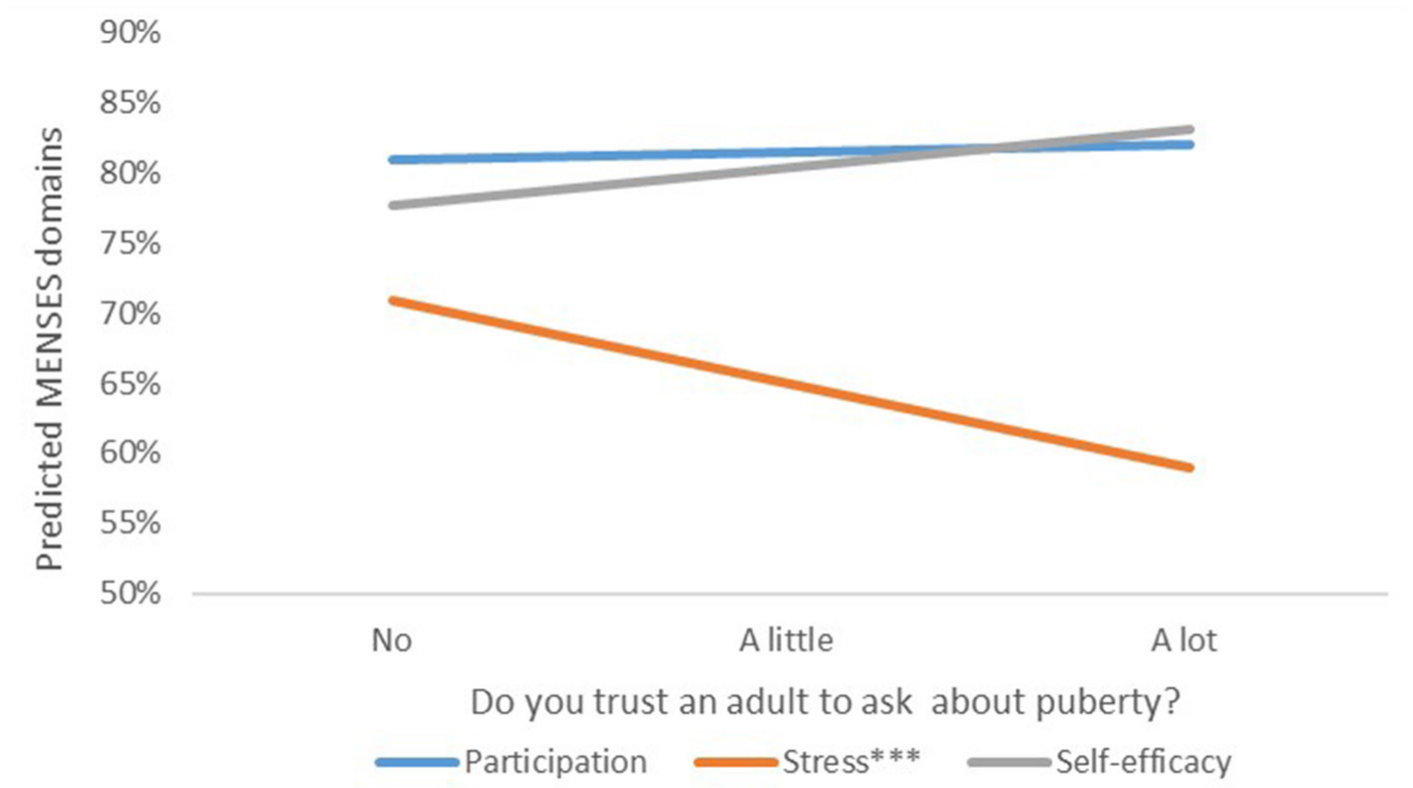
Relationship between MENSES outcomes and trusting an adult to ask about puberty. ^*^p < 0.05, ^**^p < 0.01, ^***^p < 0.001.

Among girls from low SES households, there were additional unique relationships identified. Low SES girls who reported knowing what their period was when they had it for the first time, showed significantly stronger engagement and self-efficacy, and lower stress compared to girls that did not (Table 11). These relationships were not observed among girls from the high SES group (see Table 2 in Appendix 2 in Supplementary Materials). All girls who responded they trusted an adult to ask about puberty showed significantly lower stress compared to girls who did not have this adult in their life (Figure 2). When stratified by SES status, having a trusted adult they could talk to about puberty remained associated with lower stress and was statistically significant among low SES girls (see Table 3 in Appendix 2 in Supplementary Materials). Other factors, such as cohort and region, were not significant predictors for MENSES outcomes.

**Table 11:** Predicted MENSES scores by SES and knowledge level about what their period was when they had it for the first time.

**When you had your first menstruation, did you know what it was?**	**Low SES**		
	Engagement^**^ %	Stress^**^ %	Self-efficacy^**^ %
No	73	71	75
A little	78	65	79
A lot	83	60	82

* *p < 0.05*,

** *p < 0.01*,

*** *p < 0.001*.

## Discussion

We See Equal was a small school-based gender equality program in 47 public schools across three urban centers in Mexico City, Puebla and Mérida, Mexico. This study utilizes program monitoring data to explore relationships between MHH and puberty knowledge and menstruation-related school engagement, stress and self-efficacy (MENSES) outcomes among menstruating girls in 5th and 6th grade before and after program implementation. This study contributes to understanding how early school-based MHH literacy interventions can improve girls' educational experiences by increasing school participation, reducing stress and improving self-efficacy among VYA girls who are managing their periods at school.

There are limitations to the interpretation of the data. Results are descriptive; it is not possible to draw any causal inferences about the impact of the program on MENSES outcomes. The sample is a random sample of 5th and 6th grade girls from the 47 WSE schools, not a representative sample of girls from Puebla, Mexico City, and Mérida. Additionally, the average age of menarche in Mexico is unknown. Girls may have felt uncomfortable talking about their period during surveys and chosen not to report their menstrual status. Sampling enough girls in this age group who had managed their periods while in school was challenging, though this analysis provides estimates of girls in this age group who are menstruating and can inform future M&E planning. Results should be interpreted with caution, as the study is underpowered to detect changes in MENSES outcomes across and between multiple subgroups. Finally, in-depth menstruation and puberty knowledge taught in classrooms through WSE, such as tracking the menstrual cycle, understanding menstruation and fertility, and discussion of normal physical discomforts, may have more effect on MENSES outcomes, but were not captured in the KAP results or analyzed in this study.

Previous studies have examined psychosocial outcomes and MHH as a way to measure effectiveness of MHH programming (62, 63), in particular measuring self-efficacy using existing self-efficacy scales (33, 47, 64). Results from our study support the hypothesis that increasing MHH literacy significantly improves girls' menstruation-related stress and self-efficacy, though not school engagement (Figure 1). The lack of invariance of the engagement domain requires exploring further relationships, a recent psychometric study identifies the MENSES constructs and the items that are most strongly correlated (61). The engagement and stress domains measure girls' experiences at school in such a unique way that further research is needed to determine whether both domains might collectively measure one construct related to well-being and social support. Future studies should incorporate causal research in order to better understand the impact of different combinations of MHH strategies on MENSES outcomes in Mexico, particularly in terms of dosage and cost-effectiveness.

We examine the existence of MENSES gaps by level of MHH knowledge at baseline and the extent to which gaps reduce by endline. Results suggest that increased MHH knowledge through WSE programming has contributed to closing engagement and stress gaps, especially for girls from low SES, consistent with other studies (19, 65, 66) (Tables 9, 10). Despite these improvements, work is needed to improve self-efficacy for girls with low MHH knowledge, especially girls from low SES households. Our results suggest that practitioners must continue to assess the design and delivery of their MHH programs to improve desired MHH outcomes for all girls. Examination of the individual items within each MENSES domain lend itself to consider practical school MHH interventions. Between two-thirds to three-quarters of menstruating girls consistently reported worrying about pain, getting their period unexpectedly, bloodstains, and using the school toilets (Appendix 1 in Supplementary Materials). At endline, 60% of girls still worried about using the bathroom on days when they had their period. Further exploration is required to understand what the WASH concerns may be in each context and added investments that are required, but low cost simple fixes such as locks and hooks on the doors, covered trashcans in stalls, mirrors and adequate light can alleviate some of the added burdens that girls experience using bathrooms during their menstrual period (6, 8, 67). The large proportion of girls experiencing anxiety on a monthly basis suggests that beyond education, a combination of basic MHH interventions could vastly improve girl's wellbeing (11, 46). In addition to addressing WASH concerns, ensuring schools have a supply of pads, extra school uniforms, pain relief methods, as well as an easy and discrete mechanism for girls to access these services (7, 9, 10, 36, 42, 47, 64, 68) could provide peace of mind and tangible support during this new life stage. Creating comprehensive MHH programs should include strategies that further enable behavior change and reinforce MHH information, such diversifying forms of support (69), ensuring safe and functional infrastructure (8, 19), and creating linkages to youth-friendly health services (70–72).

Girls' socioeconomic status was an important factor for MHH literacy and MENSES outcomes, as well as the associations between them. A study reviewing data across eight LMIC's found that lower income households were less likely to have access to safe spaces that lock where women could manage their periods, increasing lower SES women's risk of experiencing GBV (19). While our analysis did not focus on MHM spaces, or WASH in schools, teaching girls the practicalities of menstrual hygiene without providing safe, clean and adequate infrastructure may negate or minimize any improvements in stress or self-efficacy achieved through information alone. Girls with less economic resources may still experience increased stress attempting to practice a behavior without the proper enabling environment. In our study, girls from the low SES group demonstrated higher menstruation-related stress and less menstruation-related self-efficacy before and after the program, though there was a slight improvement in stress at endline. Further, high SES girls consistently had higher levels of MHH knowledge compared to their low SES peers, though low SES girls experienced larger gains at endline (Table 8), most notably knowing what puberty was (24 vs. 18% change) and learning about menstruation in school (19 vs. 14% change). These findings suggests that girls from lower income households may rely more on schools for critical and timely MHH and puberty education, while those from higher SES families have access to the information from other sources.

Beyond the provision of accurate and practical MHH information, having a trustworthy adult that girls can turn to arose as a key factor for MENSES outcomes. While the WSE program did not increase the proportion of girls who reported having a trusted adult they could talk to about puberty, among girls in the low SES group, having said adult was significantly associated with lowered menstruation-related stress compared to girls who did not. Though adolescence is a time that children seek more acceptance from peers (73, 74), this finding aligns with previous studies highlighting the desire of adolescents to communicate with their parents or caretakers about these topics (75). Additionally, it highlights the importance of in-service teacher training to ensure that they can sensitively provide information to their students who cannot have those critical conversations at home (3, 76). The need to provide such social-emotional support alongside puberty information may be even more acute among girls living in more impoverished families. As SES was crudely measured using the proxy of computer ownership, it likely oversimplifies the relationship between household resources, MHH knowledge, and MENSES outcomes. Overall, the associations between having a trusted adult and reduced stress, as well as the individual items responsible for decreases in stress and increases in self-efficacy (Table 4; Figure 2), suggest that the WSE program not only improved MENSES outcomes through increasing information, but importantly it improved social support among these students. Openly discussing menstruation with peers may increase feelings of support among girls simply by reducing the stigma of menstruation (67), while practical information, such as how to dispose of menstrual pads and how to track their period, led girls to feel empowered in their own abilities.

Results from this study support existing recommendations to initiate CSE and puberty education before girls reach menarche (29). Simply knowing what menstruation was before experiencing menarche was associated with lower levels of stress and higher self-efficacy and engagement among girls in WSE (Table 11). Though puberty and menstruation knowledge among this population of VYA girls was high at baseline, global research has documented that many girls learn about their period and puberty after they experience menarche (77). Among the entire population of girls surveyed in WSE (*n* = 900), 69% knew of puberty vs. the 80% who knew of puberty among those who already had their period (Table 1). We observed differences between reported MHH and puberty knowledge between the sub-population of girls who had their first period and the aggregate group. Knowledge levels are relatively high in learning about puberty at school among the entire female population (92%) and among menstruating girls (99%), compared to learning about menstruation at school (76%) among the aggregated group. Additionally, there were larger increases at endline among menstruating girls who reported learning about menstruation at school compared to the entire female sample (10 vs. 6%). The comparison of these data points suggests a disconnect between menstruation and puberty education among girls in WSE schools, with the potential that some teachers are omitting MHH information during puberty lessons or only sharing details with the girls they believe require it. This points to a need to expand MHH teacher training and monitoring to ensure teachers have the information, skills and confidence to elaborate on the topic for all students in the classroom (78).

We See Equal is a puberty education program with strong linkages to gender equality and empowerment. WSE aimed to not only increase MHH literacy and puberty knowledge, but also to create empathy between girls and boys by demonstrating both their shared and unique social and emotional transitions during puberty. Future research should examine the benefit of gender-equality focused education on girls' school experiences, particularly menstruation-related self-efficacy and school engagement, which may be more influenced by class lessons that leverage gender equality to empower girls (64). Similarly, this study did not examine the relationship between MENSES outcomes and male student knowledge, gender attitudes, or empathy for girls who are experiencing this change. Understanding how MHH literacy among boys influences girls' MENSES outcomes, especially items related to social support, would further bolster the need to involve boys and men (79) to create truly gender transformative programs that advance gender equality. Current SEP curricula focuses on biology and human reproduction, which while important, may not improve the greater wellbeing of girls and boys as they pass through this life stage. Improving the study designs of future programs would allow practitioners to make robust statements about how these programs change child-level outcomes and may have more gravitas when advocating to education authorities for improved curricula in schools and teacher training.

The analysis of We See Equal monitoring data lend themselves to concrete suggestions for future strategies to improve the overall quality of MHH programs. The finding that, girls with a trusted adult they can ask about puberty also have lower menstruation-related stress, indicates a necessity for greater parent and caretaker involvement in these programs. Parents and caretakers need MHH and puberty education themselves (80), as well as the soft skills to have these conversations with their children. Teachers may attempt to fill this gap, especially for boys and girls from lower SES groups, but teachers require additional training to address the social-emotional elements of the topic (78). Leaving out parents and caretakers in these programs contributes to the cycle of worse health and education outcomes for poorer families and children.

Future programs that aim to improve MHH literacy, MENSES outcomes and puberty education should focus on locations with lower-income populations, intervening before puberty onset, and piloting new pedagogy practices to increase social support and meet girls' needs. WSE took place in urban centers in Mexico with the majority of girls having high levels of MHH knowledge at baseline. However, girls from higher SES had more knowledge before and after the program, suggesting that these girls already had access to the information outside of schools. WSE programming may have more impact among populations with less economic resources that rely more on schools and teachers for puberty education. At baseline, 15% of girls in grades 5 and 6 reported having reached menarche, and over a quarter of them did not know what their period was the first time they had it (Table 5). This finding implies that schools are already late in providing this information to students and should consider initiating age-appropriate CSE in the 4th grade. Finally, several observations from the analysis suggest that one of the benefits of WSE was that puberty and menstruation education reduced stress and increased self-efficacy by improving girls' social support. WSE was designed to not only provide accurate and timely information to VYAs, but also provide teachers the skills and resources to conduct dynamic sessions that facilitated discussion and interaction among classmates. Future programs should explore ways to expand on this strength, potentially piloting alternative modalities that allow girls and boys to explore these topics in-depth among same-sex peers before engaging with mixed sex classrooms, building a sense of safety and confidence prior to delving into shared adolescent experiences and issues (47).

Practitioners cannot ignore the fact that this paper focuses on activities that occurred pre-COVID 19 and many of the recommendations for future programming and research must take into account current school closures and the future unknown “new normal.” From the outset, girls' reenrollment rates are predicted to drop when schools reopen and girls face harsher outcomes as a result of being out of school, families losing economic resources, and limitations to accessible health services (81). UNESCO reported that in 2021 many learners lost access to CSE and girls across the globe are less likely than boys to have the resources to participate in virtual learning, more likely to become responsible for household tasks, and experience higher feelings of stress and anxiety resulting from the pandemic (82). Trends already hint at the realization of our worst fears for girls, as the impacts of the global pandemic are showing increases in assault, domestic, and sexual violence in Mexico (22). The education and health sector, including governments and civil society, will need to adapt menstrual health and puberty education with low-tech or no-tech methods in order to reach families with this critical information (83).

If Mexico wants to achieve its goal to reduce adolescent pregnancy among 15-19 year olds and eliminate it among VYA (52), improving menstrual health literacy is a good start. It will require going beyond the current curricular components of puberty education in primary schools which focus on biology, recognizing physical changes in puberty, and linking puberty with the “ability to reproduce” (58). The need for comprehensive sexuality education, including information about puberty, menstruation and gender is critical in primary school and especially among girls who come from lower income households. Program that improve menstrual health literacy have the potential reduce menstruation-related stress and increase self-efficacy, thereby improving the quality of girls' educational experiences. Many teachers are unprepared to provide this information and training on these topics is not readily available. As these subject are rarely monitored or assessed, updating education policies to include these topics and monitor their coverage (43, 84) will be imperative for affecting change at scale. In Mexico, girls and adolescents have high rates of pregnancy, gender-based violence and young sexual debut, especially among girls who are out of school. Educating VYA and empowering them with this information at earlier ages could have longer term impacts on their future health and education.

## Data Availability Statement

The raw data supporting the conclusions of this article will be made available by the authors, without undue reservation.

## Ethics Statement

The studies involving human participants were reviewed and approved by Save the Children US Ethics Review Committee. Written informed consent to participate in this study was provided by the participants' legal guardian/next of kin.

## Author Contributions

JL provided technical support to the WSE project, including the formative research design used to adapt the Choices materials. She worked with country staff to design the survey KAP assessment questions and methods and worked on the interpretation of the analysis results for the research paper submitted. JH and JL co-developed the MENSES tool. JH reviewed data analysis and methods for this research study, contributed significantly to the literature review, and developed key conceptual arguments for the manuscript. PM led data analysis and interpretation for the research study, and has supported the psychometric analysis of the MENSES tool and its improvements over time, providing strategic guidance to evaluation methods for WSE. SV led the research, design and program operations of We See Equal in Mexico. She headed the Choices adaptation process and was intimately involved in the development of the KAP survey as well as ensuring data quality throughout program implementation. SV provided critical contextual background information, as well as supported in the interpretation of results, future research and programming needs having a perspective most closely tied to the field. All authors contributed to the article and approved the submitted version.

## Funding

Procter & Gamble funded and continues to fund the We See Equal program in Mexico (Grant # 84005101). This includes staff time and operational costs of implementation in Mexico.

## Conflict of Interest

The authors declare that the research was conducted in the absence of any commercial or financial relationships that could be construed as a potential conflict of interest.

## Publisher's Note

All claims expressed in this article are solely those of the authors and do not necessarily represent those of their affiliated organizations, or those of the publisher, the editors and the reviewers. Any product that may be evaluated in this article, or claim that may be made by its manufacturer, is not guaranteed or endorsed by the publisher.
