# Supplementary material for: The More You Know, the Less You Stress: Menstrual Health Literacy in Schools Reduces Menstruation-Related Stress and Increases Self-Efficacy for Very Young Adolescent Girls in Mexico
Source: Front Glob Womens Health. 2022 Apr 14;3:859797. doi: 10.3389/fgwh.2022.859797 (PMC9047952; doi:10.3389/fgwh.2022.859797)
Supplement: Supplementary file 1 [file Data_Sheet_1.pdf]

Appendix1. MENSES items over time

Item	Baseline	Endline	Change over time
**Participation***			
Participated in physical education class	71%	75%	4%
Spent recess alone	9%	7%	-2%
Felt like doing homework	79%	79%	0%
If the teacher asked you to go to the board for an activity, you excuse from it	17%	17%	0%
Participated in class, just like any other day	88%	90%	2%
Missed a day of school	14%	11%	-3%
Had a difficult time concentrating in class	25%	26%	1%
Left school during the school day to change your pad	33%	29%	-4%
Had a difficult time paying attention to the teacher because you were thinking about your period	33%	24%	-8%
Answered the teacher's question even though you knew the correct answer	17%	15%	-2%
**Stress**			
Worried about your period starting while in school	75%	72%	-2.3%
Were afraid that your girl classmates would gossip about you	65%	56%	-8.3%
Worried that boys would make fun of you	62%	56%	-5.8%
Felt nervous	74%	65%	-8.3%
Worried that it would be painful	71%	63%	-7.7%
Were you afraid that you would accidentally do something that would make your period worse	68%	65%	-3.4%
Worried that you would stain your school uniform	87%	79%	-8.0%
Worried about using the school toilet	67%	60%	-7.6%
Worried about having enough water to flush the toilet	79%	74%	-5.2%
Worried about how you would dispose of your pad at school	65%	55%	-9.3%
Worried that boys may peek on you when using the toilet	61%	53%	-7.3%
Felt lonely	50%	45%	-5.0%
**Self-efficacy**			
Ask a friend for help	78%	86%	9%
Get a sanitary pad if you need one	77%	83%	6%
Ask the teacher for a sanitary pad when you need one	72%	76%	5%
Ask a friend to lend you a sanitary pad	73%	83%	10%
Use the toilet anytime	84%	85%	1%
Properly dispose of your sanitary pad in a garbage	76%	80%	4%
Wash your hands after changing your sanitary pad	93%	94%	1%
Stand in front of the class to answer a question	70%	78%	8%
Do well on an exam	78%	90%	12%
Talk to an adult if you have questions about your period	78%	81%	3%
Ask the teacher for help if you stain yourself	82%	85%	3%
Handle your period without any help	61%	67%	6%
Ask the clinic teacher for painkillers if you need them.	59%	66%	8%
Report students who bully you about menstruation to the teacher	87%	90%	3%
Stand up for yourself if you are teased about menstruation at school	83%	88%	5%

Stand up for your friend if she were being bullied about menstruation at school	88%	91%	4%
Eat sour foods when you have your period.	53%	69%	16%
Track your period to know what day your next period is coming	67%	70%	3%
**Engagement items are not reverse-coded*			

Appendix 2. Multivariate regressions

Table 1. Model 1. MENSES domains and knowledge aggregated questions

Variables	Engagement		Stress		Self-efficacy	
	Coefficient	Effect size	Coefficient	Effect size	Coefficient	Effect size
Region (Mexico City=1)	-0.013	-0.098	-0.012	-0.072	-0.012	-0.117
	(0.025)		(0.032)		(0.018)	
Region (Merida=1)	-0.014	-0.107	0.037	0.223	-0.001	-0.008
	(0.024)		(0.027)		(0.017)	
SES (computer=1)	0.037	0.280	-0.031	-0.190	0.027*	0.266
	(0.020)		(0.024)		(0.013)	
Cohort	0.002	0.012	0.026	0.158	-0.027*	-0.266
	(0.018)		(0.024)		(0.013)	
**Knowledge aggregated questions (out of 5)**	**0.037**	**0.276**	**-0.126****	**-0.770**	**0.112*****	**1.111**
	**(0.031)**		**(0.046)**		**(0.024)**	
Constant	0.741***	5.544	0.783***	4.774	0.672***	6.647
	(0.040)		(0.055)		(0.030)	
N	193		193		211	

Note: * p < .05, ** p < .01, *** p < .001

Table 2. Model 2. MENSES domains and knowing what their period was when they had it for the first time

Variables	Full sample			Low SES			High SES		
	Engagement	Stress	Self-efficacy	Engagement	Stress	Self-efficacy	Engagement	Stress	Self-efficacy
	Coeff	Coeff	Coeff	Coeff	Coeff	Coeff	Coeff	Coeff	Coeff
Region (Mexico City=1)	-0.016	-0.003	-0.02	-0.033	0.014	-0.034	-0.003	-0.013	-0.009
	-0.025	-0.032	-0.019	-0.039	-0.05	-0.027	-0.032	-0.042	-0.026
Region (Merida=1)	-0.011	0.025	0.006	-0.032	0.037	-0.012	0.012	0.014	0.024
	-0.024	-0.027	-0.017	-0.039	-0.044	-0.025	-0.03	-0.034	-0.023
SES (computer=1)	0.037	-0.034	0.032*						
	-0.019	-0.023	-0.014						
Cohort	0.007	-0.005	0.008	-0.016	-0.01	-0.003	0.027	0	0.017
	-0.015	-0.018	-0.01	-0.024	-0.028	-0.013	-0.017	-0.022	-0.015
**Knowing what their period was**	**0.024**	**-0.043****	**0.017**	**0.051***	**-0.055***	**0.034****	**-0.002**	**-0.03**	**0.001**
	**-0.014**	**-0.016**	**-0.009**	**-0.023**	**-0.027**	**-0.013**	**-0.017**	**-0.02**	**-0.013**
Constant	0.759***	0.693***	0.763***	0.783***	0.709***	0.775***	0.780***	0.640***	0.790***
	-0.031	-0.036	-0.021	-0.043	-0.053	-0.03	-0.039	-0.044	-0.024
N	193	193	211	92	92	99	101	101	112

Standard errors in parentheses

Table 3. Model 3. MENSES domains and having a trusted adult to talk about puberty

Variables	Low SES					
	Engagement		Stress		Self-efficacy	
	**Coeff**	**Effect size**	**Coeff**	**Effect size**	**Coeff**	**Effect size**
Region (Mexico City=1)	-0.014	-0.097	-0.035	-0.195	0.000	0.002
	(0.043)		(0.048)		(0.026)	
Region (Merida=1)	-0.042	-0.291	0.061	0.342	-0.018	-0.188
	(0.043)		(0.047)		(0.027)	
Cohort	0.005	0.037	-0.031	-0.176	0.007	0.076
	(0.023)		(0.027)		(0.013)	
**Having a trusted adult to talk about puberty**	**0.003**	**0.019**	**-0.130***	**-0.731**	**0.053**	**0.537**
	**(0.038)**		**(0.056)**		**(0.037)**	
Constant	0.805***	5.527	0.790***	4.449	0.748***	7.631
	(0.050)		(0.058)		(0.044)	
N	84		84		90	
